# Effect of a protein intervention during resistance training with varying training intensities on muscle outcomes in frail community-dwelling older adults: a randomized controlled trial

**DOI:** 10.1016/j.jnha.2026.100838

**Published:** 2026-04-02

**Authors:** Esmée J.M. Biersteker, Mohammed Benali, Jantine van den Helder, Jos Twisk, Michael Tieland, Peter J.M. Weijs, Josje D. Schoufour

**Affiliations:** a Department of Nutrition and Dietetics, Faculty of Health, Sport and Physical Activity, Amsterdam University of Applied Sciences, Dokter Meurerlaan 8, Amsterdam, 1067 SM, the Netherlands; b Amsterdam Movement Sciences Research Institute, VU University, Amsterdam, the Netherlands; c Amsterdam Public Health Research Institute, VU University, Amsterdam, the Netherlands; d Department of Epidemiology and Data Sciences, Amsterdam University Medical Center, Amsterdam, the Netherlands; e Institute for Physical Activity and Nutrition, School of Exercise and Nutrition Sciences, Deakin University, Geelong, Victoria, Australia; f Department of Nutrition and Dietetics, Amsterdam University Medical Center, VU University, Amsterdam, the Netherlands

**Keywords:** Frailty, Older adults, Protein intake, Protein intervention, Resistance training, Sarcopenia

## Abstract

**Objectives:**

Optimising nutritional and exercise strategies is essential to preserve muscle health and physical function in frail older adults. This study aims to investigate the effects of a protein intervention during progressive resistance training (PRT) with varying training intensities on muscle strength, muscle mass, and physical performance in frail community-dwelling older adults. We were particularly interested in whether these effects differed according to variations in habitual protein intake and resistance training intensity.

**Design:**

Secondary analysis of a randomized controlled trial.

**Participants:**

This 12-week RCT randomized 295 frail community-dwelling older adults into PRT-only or PRT with a protein intervention (PRT-Pro). Frailty was defined by receipt of in-home care services or a Tilburg Frailty Indicator score ≥5.

**Intervention:**

All participants performed under one-to-one supervision, twice-weekly full-body resistance training performed until muscle failure with varying training intensities (20–80% of one-repetition maximum (1RM)). Participants in the protein intervention group received dietary counselling and tailored daily provision of whey protein supplements (20 g) to support achieving a protein intake of 1.2–1.5 g/kg/day. To prevent over-feeding, for participants with a BMI > 30 kg/m^2^, bodyweight was adjusted using a BMI of 27.5 kg/m^2^.

**Measurements:**

Primary outcome was leg press muscle strength (1RM). Secondary outcomes included appendicular lean mass and physical performance.

**Results:**

Participants had a mean age of 73.9 ± 6.0, with 69% being female. Protein intake increased in PRT-Pro compared to PRT-only (0.4 g/kg, 95%CI: 0.3−0.5, p < 0.001). Overall, the protein intervention did not result in significantly greater gains in leg press muscle strength, appendicular lean mass, or physical performance compared to PRT-only (p > 0.05). In exploratory subgroup analyses, participants with baseline habitual protein intake <1.2 g/kg showed greater improvements in leg press strength with protein intervention (6.4 kg, 95% CI: 1.3–11.4, p = 0.013), with the largest effect observed in those <0.8 g/kg (10.9 kg, 95% CI: 1.5–20.4, p = 0.025) compared with PRT-only. Training intensity did not modify the effects of the protein intervention.

**Conclusion:**

A protein intervention during resistance training did not enhance overall muscle strength, muscle mass, or physical performance in frail older adults. The protein intervention was associated with greater strength gains in exploratory analyses among participants with lower baseline protein intake (<1.2 g/kg/day), with the most pronounced associations observed in those consuming <0.8 g/kg/day. These findings highlight the potential importance of baseline protein status, although they require confirmation in larger trials involving frail older populations.

**Study ID:**

NL-OMON54919 (at ICTRP Search Portal), date of registration: 09-02-2021.

## 1 Introduction

Frailty is a common geriatric syndrome characterised by increased vulnerability to stressors and a heightened risk of adverse health outcomes, including disability and higher rates of hospitalizations in older adults [1]. A major contributor to frailty is sarcopenia, defined as the progressive loss of skeletal muscle mass, muscle strength, and physical performance with aging [2,3]. As muscle strength plays a central role in maintaining independence and daily life activities, effective strategies to prevent or treat sarcopenia are critical for promoting healthy aging, especially in frail older adults.

Progressive resistance training is widely recognized as the most effective intervention to counteract sarcopenia, as it increases muscle protein synthesis and muscle mass, and greatly improves muscle strength and physical performance [[4], [5], [6]]. Although resistance training alone is beneficial, its effects may be further increased when protein intake is optimized [[7], [8], [9]]. However, there is a lack of consensus regarding optimal protein intake in general or during resistance training. While the recommended dietary allowance for protein intake for the general population is 0.8 g/kg bodyweight (g/kg), the PROT-AGE Study Group recommends 1.0−1.2 g/kg, and other studies advocate for at least 1.2 g/kg for older adults [[10], [11], [12], [13]]. Previous research have not consistently demonstrated that combining “optimal” protein intake with resistance training enhances the effects of resistance training on muscle mass, muscle strength, and physical performance measures [2,[14], [15], [16], [17],^18^,^19^]. Importantly, evidence suggests that protein supplementation does not augment resistance training adaptations in older adults with adequate protein intake, whereas benefits appear primarily in those with low habitual intake [2,[14], [15], [16], [17],^18^,^19^]. In this context, habitual protein intake – defined as the amount of dietary protein regularly consumed as part of one’s usual diet – may represent a critical modifier [2,[19], [20], [21]]. Individuals with higher habitual protein intake, muscle protein synthesis was already sufficiently stimulated, thereby diminishing the potential benefit of additional dietary protein intake or protein supplementation [19,22]. Consequently, further research is warranted to better understand whether the effects of resistance training differ between frail older adults with low versus high habitual protein intake, as this distinction has been underexplored in previous studies.

Other factors that may influence the additional effect of protein intake on exercise training include the specifics of the training regimen, such as various training intensities [9,20]. Higher training intensities produce more muscle damage, creating a stronger anabolic stimulus and potentially a higher protein turnover that increases the need for protein to maximize muscle protein synthesis [23]. However, older adults often encounter barriers such as fatigue, poor health, and risk of injury to perform resistance training and these issues are likely worse in frail individuals during high intensity training [24]. Moreover, resistance training performed to muscle failure has been shown to produce comparable strength gains across different training intensities in adults and healthy older adults [25,26]. Previously, we found that in frail older adults, similar improvements in muscle strength were found between training intensities and suggested that training to muscle failure may already be sufficient to elicit near-maximal adaptations [27]. Nevertheless, evidence regarding the additional effect of protein while training at different training intensities performed to muscle failure frail older adults remains scarce.

Understanding factors that modify the effects of additional protein on resistance training–induced muscle adaptations is key to identifying frail older adults most likely to benefit. This study investigated the impact of a protein intervention during resistance training at varying intensities on total protein intake, muscle mass, strength, and physical performance in frail community-dwelling older adults, and whether responses were impacted by the habitual protein intake and training intensity.

## 2 Material and methods

### 2.1 Study design

The TEAMS study is a randomized controlled trial with a two-step randomization design with three pre-specified research aims described in the study protocol, (1) to identify the optimal training intensity for improving muscle strength (described elsewhere), and (2) to evaluate the additional effect of protein during various intensities of progressive resistance training on muscle strength (the focus of the present analysis), and 3) explore the role of personal characteristics [28]. The TEAMS study consisted of a twelve-week intervention period during which all participants engaged in a progressive resistance training (PRT) at varying intensities, but in any case, trained until muscle failure. Additionally, half of the participants were randomized into a protein intervention consisting of dietary counselling and protein supplementation aiming to a daily protein intake of 1.2−1.5 gram per kilogram bodyweight. Data was collected at baseline and after twelve weeks. This study was approved by the Medical Ethics Committee (METC) of the Amsterdam University Medical Center, location VUmc, the Netherlands (NL74601.029.20; Protocol ID: NL8785 [28]. The trail was prospectively registered at ICTRP Search Portal, with study-ID NL-OMON54919 and date of registration 09-02-2021. As specified in the registry and published study protocol, the primary outcome was muscle strength (see QR codes in Supplementary file 1) [28]. This study followed the CONSORT guidelines for reporting randomized trials (Supplementary file 2).

### 2.2 Participants and randomization

Participants were recruited in Amsterdam, the Netherlands, between September 2020 and September 2023 through collaborations with local healthcare providers and organizations, as well as via advertisements in local newspapers and other media outlets. Inclusion criteria required participants to be community-dwelling older adults, aged 65 years or older, receiving in-home care services or with a Tilburg Frailty Indicator score ≥5, and a Mini-Mental State Examination (MMSE) score ≥18 [29]. The Tilburg Frailty Indicator (TFI) was a questionnaire and consisted of 15 items covering three domains: physical (8 items), psychological (4 items), and social (3 items) frailty. Each item is scored dichotomously (0 = no, 1 = yes), yielding a total score ranging from 0 to 15, with higher scores indicating greater frailty [30]. Additionally, participants needed to be physically capable and willing to engage in the intervention, and able to provide written informed consent. Exclusion criteria were an inability to understand the Dutch language, the use of total parenteral nutrition or other medical or palliative treatment interfering with the intervention, or the presence of specific medical diagnoses, such as unstable coronary heart disease, decompensated heart failure, uncontrolled hypertension, arrhythmias (e.g., heart failure NYHA>3), degenerative neurocognitive disorders or chronic obstructive pulmonary disease classified as GOLD>3 [31].

After approval of the study physician, participants underwent a two-step randomization process. First, they were randomly assigned a priori to one of 13 training intensity groups, ranging from 20% to 80% of their one-repetition maximum (1-RM) with increments of 5% (20%, 25%, 30%, 35%, 40%, 45%, 50%, 55%, 60%, 65%, 70%, 75%, or 80% of 1-RM). This randomization ensured balanced allocation across the full spectrum of training intensities. The participants in the 20% training intensity group were excluded from analysis due to the inability to train until muscle failure [27], which was considered as a protocol violation. In the second step, participants were randomized to either progressive resistance training (PRT-only) or progressive resistance training with a protein intervention group (PRT-Pro). Randomization was conducted using a computer-generated randomization sequence by an independent researcher with no involvement in participant recruitment or assessments. The allocation was concealed by the independent researcher with a password protected computer file from the participants, assessors, and individuals delivering the intervention until the start of the intervention. Participants remained blinded to their assigned training intensity, while the trainers were aware of the allocation. To minimize the risk of group interference, training sessions for the PRT-only and PRT-Pro groups were conducted separately.

### 2.3 Progressive resistance training

The progressive resistance training program was performed by all participants and consisted of twice-weekly training sessions during twelve weeks. Training sessions were conducted in small groups of 4–6 participants, with each individual receiving one-on-one supervision from a certified trainer in all training sessions to ensure safety and proper execution of exercises. Each session lasted 60 min and included a 5-minute warm-up and cool-down consisting of light aerobic exercises, such as cycling. The resistance training utilized machine-based exercises targeting major muscle groups, specifically the leg press, leg extension, chest press and lateral pull-down. Each exercise was performed for three sets until muscle failure (e.g. the inability to perform another repetition while maintaining proper form). To ensure maximal effort, trainers employed motivational strategies and closely monitoring exercise execution to encourage and stimulate participants to reach true muscle failure. Each exercise had a maximum duration of 10 min, with rest periods of 1−2 min between sets and exercises. The training intensity was prescribed based on participants' randomized training intensity. To ensure progressive overload, 1-RM reassessments were conducted at weeks four and eight. Training intensity was divided into four groups: low intensity (25–35 % 1-RM), medium-low intensity (40–50 % 1-RM), medium-high intensity (55–65 % 1-RM), and high intensity (70–80% 1-RM) to analyse whether the effects of the protein intervention depend on training intensity. A more detailed description of the resistance training program is described in the TEAMS study protocol [28].

### 2.4 Dietary protein intervention

Participants randomized to the PRT-Pro group received a dietary protein intervention targeting a daily protein intake of 1.2–1.5 g/kg bodyweight/day (g/kg). The protein intervention comprised dietary counselling combining two group sessions with five individual tele-coaching sessions led by a dietician. The dieticians provided personal nutritional counselling considering the eating habits and preferences of the participant while emphasizing optimal timing of protein consumption and achieving a protein intake of >25 grams per main meal. In addition, participants were provided as a complement to the dietary counselling with two 10 g daily protein supplements, whey protein isolate and protein-enriched orange juice, to support their daily dietary protein goals, and achieving >25 grams of protein per main meal. Participants were advised to consume the protein-enriched orange juice at breakfast and the whey protein isolate as an evening snack, to distribute their protein intake throughout the day. To prevent over-feeding, for participants with a BMI > 30 kg/m^2^, bodyweight was adjusted using a BMI of 27.5 kg/m^2^ [32].

### 2.5 Habitual protein intake

Dietary protein intake was derived from a 3-day dietary record at baseline and at twelve weeks. Participants were asked to report their consumed foods for two randomly chosen weekdays and one weekend day at home. These days could be consecutive or non-consecutive, and were collected on non-training days. Participants were asked to record each meal component and/or ingredient in grams or portions separately for six meal occasions (breakfast, morning snack, lunch, afternoon snack, dinner, and evening snack). In case of incompleteness or doubt which was the case for all dietary records, the details were retrospectively updated with the participant by a dietician. Participants with completed dietary records on at least two days were included for analysis.

Records were coded with the Dutch Food Composition database (Dutch NEVO Database, version 23) and analysed with an Amsterdam University of Applied Sciences developed syntax using IBM SPSS Statistics version 29 [33]. Additional validation was performed to identify abnormal energy intake levels (<800 or >3000 kcal/day), which were subsequently reviewed for data entry or reporting errors. Total protein intake was derived per day, per meal occasion, and then averaged over the total days. Baseline values were used to create subgroups of habitual protein intake levels (≤0.8, 0.8–1.0, 1.0–1.2, and >1.2 g/kg). Adherence to the protein intervention target of achieving a protein intake of at least 1.2 g/kg was calculated as the percentage of participants within each habitual intake subgroup.

### 2.6 Outcomes

The primary outcome of the TEAMS study was lower extremity muscle strength, assessed using the 1-RM leg press. To ensure standardization and reliability, this measurement was conducted by one trained researcher who remained blinded to the randomization. Muscle strength was further assessed using the 1-RM leg extension and handgrip strength. Muscle mass was derived from body composition measures, including appendicular lean mass (predicted muscle mass of the four limbs measured with an 8-polar multifrequency Bioelectrical Impedance Analyses Tanita MC 780) and the cross-sectional area of the rectus femoris and vastus lateralis measured by 2D ultrasound (Philips Lumify L12-4 linear array transducer) [34]. Body composition measurements were performed by trained assessors, and participants were measured in a fasted state to ensure standardized assessment conditions. Physical performance was evaluated using the 30-second chair stand test and the Short Physical Performance Battery (SPPB), with gait speed and the 5-times chair stand test also analysed as separate measures. All outcomes were assessed at baseline and at twelve weeks. Participant characteristics including age, sex, BMI (kg/m^2^), ethnicity, living situation, having malnutrition (measured with the Combined Malnutrition Screening Tool using a combination of the SNAQ, MUST, MNA-SF, and MST screening tools [35]), frailty status measured with the Tilburg Frailty Indicator or receiving in-home care services, level of education (low: secondary education, intermediate: secondary vocational education (MBO), or high: higher vocational education or university (HBO)), lifestyle behaviours such as smoking and alcohol consumption, and medical history were collected at baseline. A more detailed description of these outcomes is available in the TEAMS study protocol [28].

### 2.7 Statistical analysis

Baseline characteristics were reported as mean ± SD for normally distributed continuous variables, medians with interquartile ranges (25th – 75th percentiles) for skewed continuous variables, and frequencies and proportion for categorical variables. Linear mixed models were used in all analyses to account for the repeated measurements within subjects and to investigate the effect of the protein intervention on protein intake, muscle mass, muscle strength, and physical performance. All models included the protein intervention (yes/no) as a fixed variable, along with the baseline value of the outcome, while subjects were added as random intercept. Two different models were constructed for the relationship between protein intervention and all outcomes to account for potential confounders of sociodemographic characteristics. The first model was adjusted for age and sex, which are determinants of muscle strength and physical performance and are routinely included in aging research [36]. The second model was additionally adjusted for BMI, ethnicity, education, smoking behaviour and alcohol consumption. These variables are commonly considered key sociodemographic and lifestyle determinants in epidemiological studies and are widely used as potential confounders in analyses of health outcomes in older adults [37]. The second model was used to describe the results.

First, we examined whether the protein intervention was effective in increasing protein intake for all participants and stratified for habitual protein intake levels at baseline (≤0.8, 0.8–1.0, 1.0–1.2, and >1.2 g/kg). Differences in effect of the protein intervention on total protein intake was investigated with a high habitual protein intake (>1.2 g/kg) as reference category. Second, the overall effects of the protein intervention on muscle strength, muscle mass, and physical performance were investigated for the total study population. Next, in line with the research aims described in the study protocol, analyses exploring the role of habitual protein intake and training intensity were conducted. These analyses were not explicitly specified as stratified subgroup analyses and should therefore interpreted with caution. Due to reduced sample sizes within habitual protein intake and training intensity groups, statistical power may have been limited for these analyses. So third, to evaluate whether the effect of the protein intervention was depended on habitual protein intake or training intensity, the interaction between the intervention and habitual protein intake or training intensity were added to the analyses, and for both variables the highest group was used as reference category. Regardless of statistical significance of these interaction terms, the effects of the protein intervention were further explored in stratified analyses by habitual protein intake or training intensity. In addition, the effects of the protein intervention on muscle strength, muscle mass, and physical performance were investigated for participants not reaching a habitual protein intake of >1.2 g/kg, as these individuals are most likely to benefit from additional protein due to their insufficient baseline intake. Additional effect modification was investigated for age (categorical based on median) and sex (categorical) on the primary outcome. Beta coefficients with 95% confidence intervals (CI) were reported. A p-value of <0.05 was considered to be statistically significant. All analyses were performed using IBM SPSS Statistics (version 28) and figures were made with R studio (version 2024.04.2 + 764). The data of this study, as well as the statistical analysis plan and research protocol, is available in a data package on the Figshare repository (https://doi.org/10.21943/auas.29666096).

## 3 Results

### 3.1 Enrolment

**Fig. 1:**
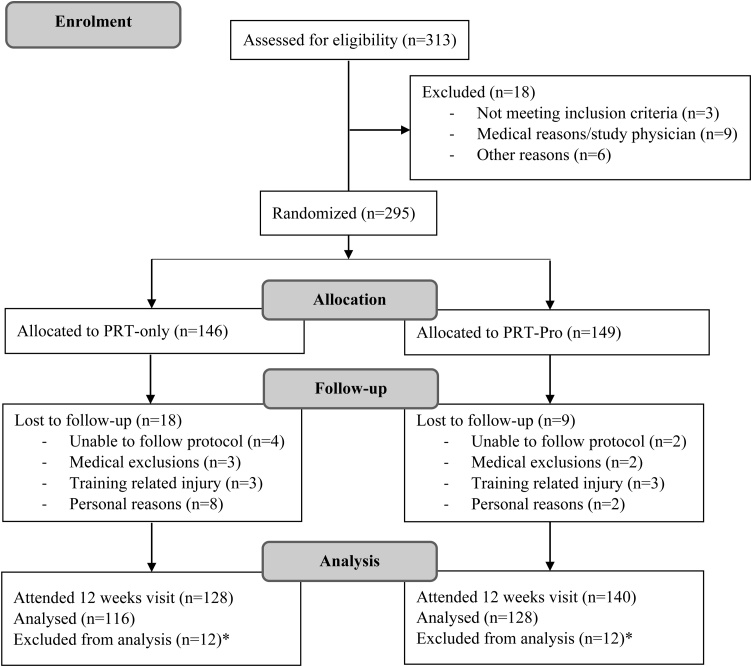
CONSORT flow diagram of participants of the TEAMS study. * Exclusion of the 20% intensity training group due to inability to achieve muscle failure within the 10-minute timeframe, which is considered a protocol violation.

A total of 295 participants were randomized into the PRT-only group (n = 146) or the PRT-Pro group (n = 149). Drop-outs were due to the inability to comply with the protocol (n = 6), medical issues (n = 5), training-related injuries (n = 6), and personal reasons (n = 10) (Fig. 1). In total, 116 participants were analysed in the PRT-only group and 128 participants analysed in the PRT-Pro group.

### 3.2 Baseline characteristics

The average age of participants was 73.9 years (SD = 6.0), with 69% being female. Most participants were of Western ethnicity (76%) and lived alone (63%). The mean baseline BMI was 27.8 kg/m^2^ (SD = 5.2), with 34% classified as having normal weight, 34% as overweight, and 29% as obese. Having diabetes was reported in 14% of the participants and 43% reported having one or more cardiovascular problems (Table 1).

**Table 1:** Baseline characteristics of participants of the TEAMS study.

Characteristics	All participants (n = 295)	PRT-only (n = 146)	PRT-Pro (n = 149)
Age	73.9 ± 6.0	74.0 ± 5.8	73.8 ± 6.2
Female, n (%)	204 (69)	103 (71)	101 (68)
Bodyweight (kg)	77.5 ± 16.1	77.4 ± 14.6	77.6 ± 17.5
Height (cm)	166.9 ± 10.0	166.1 ± 10.1	167.7 ± 9.9
BMI (kg/m^2^)	27.8 ± 5.2	28.1 ± 4.9	27.5 ± 5.4
Underweight (BMI 18.5–24.9), n (%)	0 (0)	0 (0)	0 (0)
Normal weight (BMI 25–29.9), n (%)	99 (34)	42 (29)	57 (38)
Overweight (BMI ≥ 30), n (%)	101 (34)	57 (39)	44 (30)
Obese, n (%)	86 (29)	43 (30)	43 (29)
Ethnicity, n (%)			
Western	223 (76)	106 (73)	117 (79)
Non-Western	72 (24)	40 (27)	32 (22)
Living situation, n (%)			
Independently, alone	186 (63)	98 (67)	88 (59)
Independently, together	99 (34)	45 (31)	54 (36)
Service flat	10 (3)	3 (2)	7 (5)
Frailty status (TFI)^1^			
Frail, n (%)	198 (67)	99 (68)	99 (66)
Severely frail, n (%)	24 (8)	10 (7)	14 (9)
Receiving in-home care, n (%)	73 (25)	37 (25)	36 (24)
Level of education, n (%)^2^			
Low	79 (27)	38 (26)	41 (28)
Intermediate	63 (21)	31 (21)	32 (22)
High	153 (52)	77 (53)	76 (51)
Current smoking, n (%)^3^	17 (6)	7 (5)	10 (7)
Current alcohol consumption, n (%)^4^	190 (64)	91 (62)	99 (66)
Having malnutrition, n (%)			
No malnutrition, n (%)	271 (92)	135 (91)	136 (93)
Malnutrition, n (%)	24 (8)	14 (9)	10 (7)
Morbidities, n (%)^5^			
Diabetes	40 (14)	23 (16)	17 (11)
Respiratory	41 (14)	17 (12)	24 (16)
Cardiovascular	127 (43)	61 (42)	66 (44)
Hypertension	89 (29)	46 (32)	43 (29)
Arthrosis	108 (37)	52 (36)	56 (38)
Musculoskeletal disorders	111 (38)	46 (32)	65 (44)
Protein intake (g/day)	78.2 ± 19.9	78.2 ± 21.0	78.2 ± 18.9
Protein intake (g/kg bw/day)	1.0 ± 0.3	1.0 ± 0.3	1.1 ± 0.3
Lower extremity muscle strength (kg)			
Leg press	101.0 ± 26.5	100.9 ± 26.0	101.0 ± 27.1
Leg extension	51.3 ± 17.0	51.3 ± 17.2	51.3 ± 16.8
Hand grip strength	27.8 ± 9.2	27.5 ± 9.5	28.2 ± 9.0
Appendicular lean soft tissue mass (kg)	20.5 ± 4.5	20.3 ± 4.4	20.6 ± 4.6
Cross-sectional area of the rectus femoris (cm^2^)	3.8 ± 1.6	3.9 ± 1.6	3.7 ± 1.6
Cross-sectional area of the vastus lateralis (cm^2^)	14.0 ± 4.2	13.8 ± 4.2	14.2 ± 4.2
SPPB score	9.7 ± 1.8	9.9 ± 1.7	9.5 ± 1.9
Gait speed (m/s)	1.1 ± 0.3	1.1 ± 0.2	1.1 ± 0.3
5-times chair stand test (s)	15.1 ± 4.9	14.4 ± 4.3	15.7 ± 5.4
30-seconds chair stand test (score)	11.2 ± 3.7	11.5 ± 3.5	11.0 ± 3.8

BMI, body mass index; MMSE, Mini-Mental State Examination; SD, standard deviation; SPPB, Short Physical Performance Battery; TFI, Tilburg Frailty Index.

Baseline characteristics are presented in mean ± SD for continuous variables and n (%) for categorical variables.

1 Frailty status was determined with the Tilburg Frailty Indicator (TFI) where available; TFI 5–9 = frail, TFI ≥ 10 = severely frail. Not all participants completed the TFI. For participants without TFI, study eligibility as “frail” was established a priori by receiving in-home care services. Accordingly, frail percentages are calculated among participants with TFI data only, whereas receiving in-home care is reported for participant who did not complete the TFI.

2 Education: Highest obtained educational degree. Low level of education: secondary education, intermediate level: secondary vocational education (MBO), high level: higher vocational education or university (HBO).

3 Current smoking: below 5 cigarettes per day.

4 Current alcohol consumption: below or even as 7 glasses on average per week.

5 Morbidity: The counts are reported per participant. If a participant has one or more issues within a given subcategory, it is recorded as a single occurrence. Musculoskeletal disorders contains musculoskeletal disorders as well as neuromuscular disorders and rheumatism.

### 3.3 Effects of the protein intervention on total daily protein intake

At baseline, protein intake of PRT-only was on average 1.0 ± 0.3 g/kg and PRT-Pro was on average 1.1 ± 0.3 g/kg. At follow-up, PRT-Pro consumed on average 0.4 g/kg (95% CI: 0.3−0.5, p < 0.001) more protein than the PRT-only group, reaching an average protein intake of 1.4 g/kg. For all habitual protein intake groups (≤0.8, 0.8–1.0, 1.0–1.2, and >1.2 g/kg), PRT-Pro significantly increased protein intake with 0.3 g/kg (95% CI: 0.1−0.4, p = 0.003), 0.4 g/kg (95% CI: 0.2−0.5, p < 0.001), 0.5 g/kg (95% CI: 0.3−0.6, p < 0.001) and 0.4 g/kg (95% CI: 0.3−0.6, p < 0.001), compared to the respective PRT-only group (Supplementary file 3 - Figure S1). The protein intervention goal of >1.2 g/kg was achieved by 32% of participants in the ≤0.8 g/kg group, 70% in the 0.8−1.0 g/kg group, 88% 1.0−1.2 g/kg group, and 90% in the >1.2 g/kg group. At follow-up, no between-group differences in the effect of the protein intervention on protein intake were observed between the habitual protein intake groups or the four training intensity groups.

### 3.4 Overall effects of the protein intervention during progressive resistance training

**Fig. 2:**
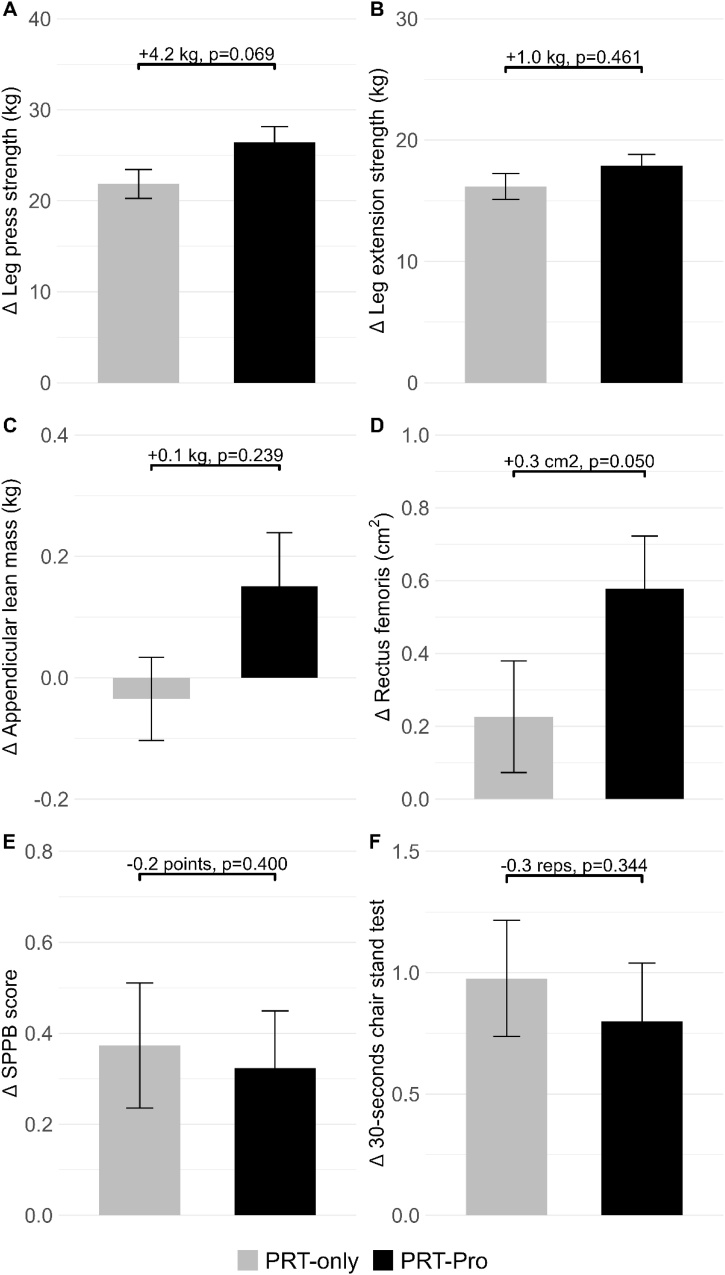
Mean change of observed values in muscle outcomes between baseline and follow-up with standard error bars in leg press strength (A), leg extension strength (B), appendicular lean mass (C), cross-sectional area of the rectus femoris (D), SPPB score (E) and 30-seconds chair stand test (F) for resistance training only (PRT-only) and resistance training with protein intervention (PRT-Pro). Linear mixed model results (β-coefficients and p-values) are presented, indicating the effect of the protein intervention adjusted for age, sex, BMI, ethnicity, education, smoking behaviour and alcohol consumption.

In the full study population, no significant additional effect of the protein intervention was found for leg press muscle strength (4.2 kg, 95% CI: −0.3−8.8, p = 0.069) (Fig. 2; Table 2, Table 3). No other significant differences were found for secondary outcomes in the total study population. No significant effect modification by age or sex was observed.

**Table 2:** Additional effects of the protein intervention (yes/no), overall and stratified by habitual protein intake at baseline.

		All participants (n = 244)	Protein intake ≤0.8 g/kg (n = 52)	Protein intake 0.8−1.0 g/kg (n = 62)	Protein intake 1.0−1.2 g/kg (n = 67)	Protein intake >1.2 g/kg (n = 58)
Outcome variable	Protein	β (95% CI)	β (95% CI)	β (95% CI)	β (95% CI)	β (95% CI)
Leg press muscle strength (kg)	Model 1	3.7 (−1.0−8.4)	**11.4 (1.1−21.8)**	2.4 (−6.1−11.0)	2.9 (−5.9−11.6)	−3.5 (−13.8−6.9)
	Model 2	4.2 (−0.3−8.8)	**10.9 (1.5−20.4)**	1.9 (−6.0−9.9)	4.0 (−5.4−13.4)	−1.8 (−12.0−8.4)
Leg extension muscle strength (kg)	Model 1	1.2 (−1.5−3.8)	**4.7 (0.2−9.3)**	1.4 (−3.3−6.1)	−0.9 (−6.5−4.8)	−0.1 (−6.3−6.2)
	Model 2	1.0 (−1.7−3.6)	4.5 (−0.1−9.0)	2.0 (−2.8−6.8)	−0.4 (−6.2−5.5)	−0.3 (−6.4−5.8)
Hand grip strength (kg)	Model 1	0.7 (−0.3−1.7)	−0.1 (−1.9−1.7)	1.7 (−0.3−3.7)	0.9 (−1.2−3.0)	1.0 (−0.8−2.7)
	Model 2	0.6 (−0.3−1.6)	0.2 (−1.6−2.0)	1.0 (−0.9−3.0)	0.5 (−1.7−2.7)	1.3 (−0.5−3.1)
Appendicular lean soft tissue mass (kg)	Model 1	0.1 (−0.1−0.3)	0.1 (−0.3−0.5)	0.3 (−0.3−0.9)	0.1 (−0.3−0.5)	−0.1 (−0.4−0.3)
	Model 2	0.1 (−0.1−0.4)	0.1 (−0.4−0.5)	0.3 (−0.3−0.9)	0.1 (−0.4−0.4)	0.1 (−0.3−0.4)
Cross-sectional area of the rectus femoris (cm^2^)	Model 1	0.2 (−0.1−0.6)	0.6 (−0.2−1.5)	−0.1 (−0.6−0.5)	0.3 (−0.4−1.1)	0.3 (−0.4−0.9)
	Model 2	0.3 (0.0−0.7)	0.6 (−0.2−1.4)	−0.1 (−0.7−0.4)	0.6 (−0.1−1.4)	0.4 (−0.2−1.0)
Cross-sectional area of the vastus lateralis (cm^2^)	Model 1	0.6 (−0.2−1.4)	**1.8 (0.3−3.3)**	0.3 (−1.3−1.9)	0.9 (−0.9−2.7)	−0.1 (−1.5−1.2)
	Model 2	0.7 (−0.1−1.5)	**1.7 (0.3−3.1)**	0.3 (−1.2−1.8)	0.6 (−1.3−2.4)	−0.3 (−1.8−1.2)
SPPB score	Model 1	−0.2 (−0.5−0.2)	−0.1 (−0.8−0.7)	−0.4 (−1.1−0.3)	0.3 (−0.4−1.0)	−0.5 (−1.2−0.1)
	Model 2	−0.2 (−0.5−0.2)	−0.1 (−0.8−0.7)	−0.4 (−1.1−0.3)	0.3 (−0.4−1.1)	**−0.7 (−1.4 to −0.1)**
Gait speed (m/s*10)	Model 1	−0.2 (−0.6−0.3)	0.3 (−0.3−1.0)	0.1 (−0.7−1.0)	−0.3 (−1.3−0.7)	−0.5 (−1.4−0.3)
	Model 2	−0.2 (−0.6−0.2)	0.3 (−0.3−0.9)	0.2 (−0.6−1.0)	−0.5 (−1.5−0.5)	−0.4 (−1.3−0.4)
5-times chair stand test (s)	Model 1	−1.7 (−4.5−1.2)	0.1 (−1.3−1.5)	1.2 (−0.4−2.9)	−0.1 (−1.6−1.4)	−0.2 (−1.3−0.8)
	Model 2	−1.9 (−4.7−1.0)	0.2 (−1.2−1.6)	0.8 (−0.8−2.4)	−0.1 (−1.7−1.5)	0.1 (−0.9−1.1)
30-seconds chair stand test (score)	Model 1	−0.2 (−0.9−0.4)	−0.2 (−1.5−1.2)	−0.4 (−1.6−0.7)	−0.1 (−1.3−1.1)	−0.5 (−1.8−0.8)
	Model 2	−0.3 (−0.9−0.3)	0.1 (−1.1−1.4)	−0.4 (−1.5−0.8)	−0.3 (−1.5−1.0)	−0.7 (−1.9−0.5)

SPPB, Short Physical Performance Battery.

Data were analysed using Linear Mixed Models to account for repeated measures within subjects with the protein intervention (yes/no) and baseline values as fixed variables. Subjects were added as a random intercept.

The number of participants included in the analyses is reported for the total study population and stratified by habitual protein intake (5 missing values). Participant numbers vary slightly across outcomes.

Model 1 was adjusted for age and sex. Model 2 was adjusted for age, sex, BMI, ethnicity, education, smoking behaviour and alcohol consumption. Bold results have a p-value < 0.05.

**Table 3:** Effects of the protein intervention (yes/no), overall and stratified by training intensity.

		All participants (n = 244)	Low intensity (n = 57)	Medium-low intensity (n = 64)	Medium-high intensity (n = 63)	High intensity (n = 60)
Outcome variable	Protein	β (95% CI)	β (95% CI)	β (95% CI)	β (95% CI)	β (95% CI)
Leg press muscle strength (kg)	Model 1	3.7 (−1.0−8.4)	7.1 (5.2−19.5)	4.2 (−2.9−11.3)	−1.3 (−10.8−8.2)	4.2 (−4.4−12.8)
	Model 2	4.2 (−0.3−8.8)	8.4 (−3.6−20.4)	**7.3 (0.8−13.7)**	−2.6 (−11.9−6.6)	6.8 (−1.0−14.6)
Leg extension muscle strength (kg)	Model 1	1.2 (−1.5−3.8)	2.9 (−2.2−8.0)	4.0 (−1.4−9.4)	−4.5 (−10.1−1.0)	0.5 (−4.7−5.7)
	Model 2	1.0 (−1.7−3.6)	2.8 (−2.1−7.7)	5.1 (−0.4−10.5)	**−6.2 (−11.8 to −0.6)**	0.6 (−3.9−5.1)
Hand grip strength (kg)	Model 1	0.7 (−0.3−1.7)	0.9 (−1.2−2.9)	1.0 (−1.1−3.0)	0.1 (−1.9−2.0)	1.5 (−0.2−3.3)
	Model 2	0.6 (−0.3−1.6)	0.9 (−1.1−3.0)	1.1 (−0.9−3.1)	−0.1 (−1.9−1.7)	**2.2 (0.6−3.8)**
Appendicular lean soft tissue mass (kg)	Model 1	0.1 (−0.1−0.3)	0.2 (−0.2−0.6)	0.1 (−0.4−0.4)	0.1 (−0.3−0.4)	−0.1 (−0.7−0.5)
	Model 2	0.1 (−0.1−0.4)	**0.4 (0.1−0.8)**	−0.1 (−0.4−0.4)	0.1 (−0.2−0.4)	0.2 (−0.4−0.8)
Cross-sectional area of the rectus femoris (cm^2^)	Model 1	0.2 (−0.1−0.6)	0.1 (−0.7−0.7)	0.2 (−0.6−0.9)	0.4 (−0.3−1.1)	0.3 (−0.3−0.9)
	Model 2	0.3 (0.0−0.7)	0.1 (−0.7−0.8)	0.1 (−0.6−0.8)	0.4 (−0.2−1.0)	0.6 (−0.1−1.2)
Cross-sectional area of the vastus lateralis (cm^2^)	Model 1	0.6 (−0.2−1.4)	−0.3 (−2.2−1.6)	**1.5 (0.3−2.6)**	0.1 (1.6−1.7)	0.2 (−1.4−1.7)
	Model 2	0.7 (−0.1−1.5)	−0.1 (−1.9−1.8)	**1.5 (0.3−2.7)**	−0.3 (−1.9−1.3)	−0.2 (−1.7−1.3)
SPPB score	Model 1	−0.2 (−0.5−0.2)	0.3 (−0.4−1.0)	−0.5 (−1.1−0.2)	−0.1 (−0.7−0.7)	−0.2 (−1.0−0.5)
	Model 2	−0.2 (−0.5−0.2)	0.3 (−0.5−1.0)	−0.5 (−1.1−0.2)	−0.1 (−0.7−0.7)	−0.3 (−1.0−0.5)
Gait speed (m/s*10)	Model 1	−0.2 (−0.6−0.3)	0.4 (−0.5−1.2)	−0.5 (−1.4−0.4)	−0.05 (−1.3−0.4)	0.2 (−0.6−1.1)
	Model 2	−0.2 (−0.7−0.2)	0.1 (−0.8−0.9)	−0.5 (−1.4−0.3)	−0.6 (−1.4−0.3)	0.3 (−0.4−1.1)
5-times chair stand test (s)	Model 1	−1.7 (−4.5−1.2)	−0.7 (−2.2−0.8)	−2.7 (−8.1−2.7)	−5.3 (−11.3−0.8)	1.5 (5.6−8.7)
	Model 2	−1.9 (−4.7−1.0)	−0.6 (−2.1−0.9)	−2.8 (−8.0−2.4)	−6.0 (−11.9−0.1)	2.6 (−4.2−9.5)
30-seconds chair stand test (score)	Model 1	−0.2 (−0.9−0.4)	**1.2 (0.2−2.2)**	−0.9 (−2.0−0.1)	−0.9 (−2.3−0.4)	0.1 (−1.2−1.4)
	Model 2	−0.3 (−0.9−0.3)	1.1 (−0.1−2.2)	−0.8 (−1.9−0.3)	−1.2 (−2.5−0.2)	0.1 (−1.2−1.5)

SPPB, Short Physical Performance Battery.

Data were analysed using Linear Mixed Models to account for repeated measures within subjects with the protein intervention (yes/no) and baseline values as fixed variables. Subjects were added as a random intercept.

The number of participants included in the analyses is reported for the total study population and stratified by training intensity. Participant numbers vary slightly across outcomes.

Model 1 was adjusted for age and sex. Model 2 was adjusted for age, sex, BMI, ethnicity, education, smoking behaviour and alcohol consumption. Bold results have a p-value < 0.05.

### 3.5 Effect of the protein intervention stratified by habitual protein intake levels

**Fig. 3:**
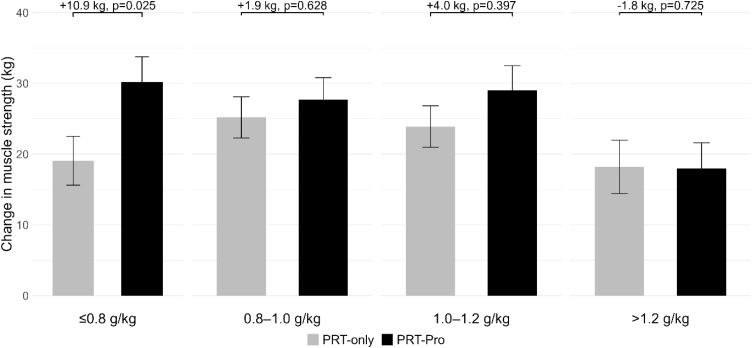
Mean change of observed values between baseline and follow-up with standard error bars in leg press muscle strength for resistance training only (PRT-only) and resistance training with protein intervention (PRT-Pro), shown for all participants and stratified by habitual protein intake. Linear mixed model results (β-coefficients and p-values) are presented, indicating the effect of the protein intervention adjusted for age, sex, BMI, ethnicity, education, smoking behaviour and alcohol consumption.

Participants increased leg press muscle strength in all habitual protein intake groups (≤0.8, 0.8−1.0, 1.0−1.2, >1.2 g/kg; Fig. 3; Supplementary file 3 - Figure S2). The protein intervention resulted in significant improvements in leg press muscle strength during resistance training in participants with an habitual intake below the recommended habitual intake of 1.2 g/kg (6.4 kg, 95% CI: 1.3–11.4, p = 0.013, n = 181) and an increase in leg extension (2.5 kg, 95% CI: −0.2–5.2, p = 0.065). There was a significant interaction effect between protein intervention and habitual protein intake groups for muscle strength (p = 0.038), but not for muscle mass and physical performance outcomes (p ≥ 0.05). For participants with a low habitual protein intake (≤0.8 g/kg, n = 52), PRT-Pro resulted in a significant improvement in leg press (10.9 kg, 95% CI: 1.5−20.4, p = 0.025) and a greater increase in leg extension strength (4.5 kg, 95% CI: −0.1−9.0, p = 0.051) compared to PRT-only (Table 2; Fig. 3; Supplementary file 3 - Figure S2). No significant between-group differences in effect of the protein intervention were found for other outcomes such as handgrip strength, muscle mass, and physical performance between the four habitual protein intake groups (Table 2). Due to reduced sample sizes within habitual protein intake groups, statistical power may have been limited for these analyses.

### 3.6 Effect of the protein intervention in different training intensity groups

**Fig. 4:**
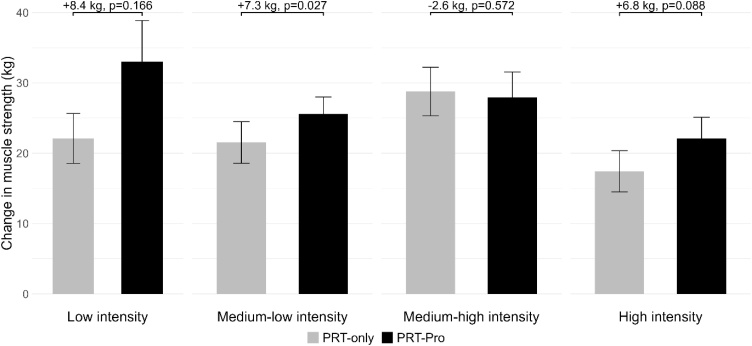
Mean change of observed values between baseline and follow-up with standard error bars in leg press muscle strength for resistance training only (PRT-only) and resistance training with protein intervention (PRT-Pro), shown for all participants and stratified by training intensity. Linear mixed model results (β-coefficients and p-values) are presented, indicating the effect of the protein intervention adjusted for age, sex, BMI, ethnicity, education, smoking behaviour and alcohol consumption.

Participants increased leg press muscle strength in all four intensity groups (low, medium-low, medium-high, and high intensity; Fig. 4; Supplementary file 3 - Figure S3). There was no significant interaction effect between protein intervention and training intensity groups for all outcomes (p ≥ 0.05). When stratified by training intensity, PRT-Pro resulted in a difference in leg press strength with 8.4 kg (95% CI: −3.6−20.4, p = 0.166, n = 57), 7.3 kg (95% CI: 0.8−13.7, p = 0.027, n = 64), −2.6 kg (95% CI: −11.9−6.6, p = 0.572, n = 63) and 6.8 kg (95% CI: −1.0−14.6, p = 0.088, n = 60), for low, medium-low, medium-high, and high training intensity respectively compared to PRT-only (Table 3). Due to reduced sample sizes within training intensity groups, statistical power may have been limited for these analyses.

## 4 Discussion

This randomized controlled trial demonstrated that the protein intervention was successful in increasing protein intake in frail community-dwelling older adults. Overall, the protein intervention did not result in a significantly greater increase in muscle strength, muscle mass or physical performance. In participants with habitual protein intake below the recommended 1.2 g/kg, the protein intervention significant improved leg press muscle strength by 6.4 kg. Notably, participants with a low habitual protein intake level of ≤0.8 g/kg experienced the greatest benefit of the protein intervention on muscle strength, showing increases of approximately 10% in leg press and leg extension muscle strength. We did not find clear differences in the effect of the protein intervention during resistance training performed to muscle failure across varying training intensities.

The protein intervention was highly effective in increasing protein intake among frail community-dwelling older adults. By combining individualized dietary counselling with the provision of protein supplements, participants achieved an average increase of 0.4 g/kg, reaching an average protein intake of 1.4 g/kg. The success of the intervention can be attributed to the use of trained dieticians who were able to adapt strategies to the individual’s needs, the structured follow-up sessions that encouraged continued adherence, and the daily provision of protein supplements and/or supplemented foods. Achieving such a high protein intake in frail older adults is noteworthy, as this group typically faces considerable barriers to meeting nutritional recommendations, such as reduced appetite [38]. Among participants with low habitual protein intake of ≤0.8 g/kg, 32% achieved the target of >1.2 g/kg. In this subgroup, which included a relatively high proportion of overweight and obese participants, protein targets were set more conservatively, and greater emphasis was placed on protein-enriched food products to minimize the impact on total energy intake. In line with our findings, a study that also combined dietary counselling with the provision of protein products reported similar high protein intake levels after their intervention, averaging 1.5 g/kg [39]. Other studies with dietary counselling or protein supplementation also reported significant increases in protein intake in older adults, where average protein intakes exceeded 1.2 g/kg [40,41]. Taken together, these findings underscore that combining dietary counselling and protein supplementation is a very effective strategy to increase protein intake and may be necessary to overcome nutritional challenges which is of particular relevance in frail older adults.

In the full study population, a protein intervention combined with resistance training did not result in a significantly greater increase in muscle strength when compared with resistance training alone. Participants in both groups achieved substantial strength gains through the resistance training, which may have limited an additional improvement from the protein intervention [27]. Previous studies have yielded inconsistent results regarding the combined effect on muscle mass, muscle strength, or physical performance, with some reporting no additional benefits [14,17,19], while others did find additional benefits [2,7,[14], [15], [16]]. These discrepancies may be explained by differences in populations across studies and reviews [42]. While some reviews focused exclusively on healthy older adults, others included frail, sarcopenic, or obese individuals, with considerable variation in age ranges across studies [19]. The absence of statistically significant effects of the combined protein intervention and resistance training on muscle mass and physical performance may reflect ceiling effects due to the high baseline functional status of participants, as well as substantial variation between individuals.

In line with our hypothesis, this study observed that the effect of the protein intervention on muscle strength was more pronounced in frail participants with low habitual protein intake (<1.2 g/kg), mainly driven by the group with a habitual intake ≤0.8 g/kg. A plausible explanation is that individuals with low protein intake may lack sufficient essential amino acids to stimulate muscle protein synthesis following resistance training and additional protein can overcome this anabolic resistance [7]. The current recommended dietary allowance of 0.8 g/kg represents the minimum intake needed to meet basic protein needs [13]. However, previous studies suggest that higher intakes – around 1.2 g/kg or more – may be necessary to support muscle maintenance and function in older adults, particularly in frail older adults and/or during resistance training [[10], [11], [12], [13]]. Other studies investigating the additional effect of protein during resistance training were already consuming a protein intake of >1.0−1.2 g/kg and most studies have not accounted for habitual protein intake [2,19,43]. To our knowledge, this is the first trial in frail older adults performing resistance training to suggest that the effect of additional protein was modified by habitual protein intake. However, the observed larger strength gains in participants with lower baseline protein intake should be interpreted cautiously, as habitual protein intake was self-reported and misreporting could have led to misclassification around the ≤0.8 g/kg/day cut point. In addition, the lack of consistent effects for other muscle outcomes further underscores that this finding should be considered exploratory and hypothesis-generating. More research is needed for other outcomes with larger sample sizes of older adults with a habitual protein intake of below, or well below, 1.2 grams of protein per kg bodyweight.

We hypothesized that higher training intensities, which provide a stronger anabolic stimulus, would enable protein intake to further enhance muscle protein synthesis and potentially augment muscle mass, muscle strength and physical performance. However, our findings suggests that training intensity appeared to not substantially influence the additional effect of the protein intervention during resistance exercise training. One possible explanation is that all participants trained to muscle failure, thereby standardizing effort and fatigue across intensity groups and limited the potential for a protein intervention to confer further benefits at higher intensities. Consistent with this interpretation, previous results in our TEAMS study found that in frail older adults, similar improvements in muscle strength were found between training intensities and suggested that training to muscle failure may already sufficient to elicit near-maximal adaptations [27]. This could limit the detectable benefit of additional protein intake. Importantly, in this study, we deliberately chose to isolate one training variable – training intensity – while standardizing other key components of the resistance training, including frequency, training until muscle failure or effort, supervision, rest intervals, and progression. This design allows a focused examination of training intensity, but is does not permit conclusions whether protein supplementation would exert a different effect under conditions where other training variables are manipulated. In addition, the subgroup analyses may have been underpowered to detect small differences between intensity groups; therefore, the absence of statistically significant differences should not be interpreted as evidence of no effect. Another possible explanation is that short-term strength gains during resistance training are largely driven by neural adaptations – such as increased motor unit recruitment and firing rate – rather than by muscle hypertrophy [44,45]. Since these neural mechanisms are stimulated effectively by resistance training alone, even at varying intensities when training to muscle failure, additional protein intake may not further enhance strength outcomes in the short-term [44,45]. Longer intervention periods may be required to determine whether protein supplementation can augment hypertrophic adaptations and translate into greater strength gains during resistance training at higher intensity in this population, ultimately contributing to the prevention of muscle wasting and the onset of sarcopenia.

This randomized controlled trial, conducted partly in the COVID pandemic and involving a large sample of frail community-dwelling older adults, had several notable strengths, for example a low dropout rate, one-to-one supervision during training sessions that guaranteed training until muscle failure, and individualized nutritional support. Participants trained across a wide range of intensities enabling a more nuanced evaluation of the effectiveness of resistance training beyond traditional high intensity protocols. Nonetheless, several limitations should be considered. Protein intake was assessed using a self-reported three-day dietary record, which may be subject to reporting bias. This method has been validated and is considered suitable for estimating protein intake in older adults, demonstrated by acceptable correlation with objective urinary biomarkers [33,46]. However, in this vulnerable population, misreporting may have influenced the classification of participants into habitual protein intake group and should therefore be taken into account when interpreting the subgroup findings. This study specifically targeted frail community-dwelling older adults, which is a heterogeneous population that included individuals with mild cognitive impairments, as well as those with obesity or malnutrition. In addition, participants had a relatively high functional status, which may have limited the potential for improvement in some physical performance outcomes. Moreover, the subgroup analysis of habitual protein intake levels and training intensities may have been underpowered to detect true differences between the resistance training group and the protein intervention group. The presence of multiple subgroup comparisons increases the risk of type I error, and the observed findings should therefore be interpreted with caution. Another limitation is the exclusion of the 20% intensity training group due to difficulties in achieving muscle failure within the 10-minute session timeframe. This underscores the practical challenges and limited feasibility of very low-intensity resistance training in both research and clinical contexts.

Achieving a higher protein intake is of particular importance in a frail population, as adequate protein is crucial for preserving muscle mass and strength, and has been associated with a lower risk of sarcopenia, frailty and slower functional decline [10,47]. This study demonstrated that a protein intervention combining individualized dietary counselling every two weeks with daily 20 g protein supplementation was highly effective in increasing protein intake, even among participants whose habitual intake was below or substantially below 1.2 g/kg. Importantly, in participants with low habitual protein intake who received the protein intervention, this study observed an approximately 10% greater increase in leg press (10.9 kg) and leg extension strength (4.5 kg) compared with training-only. These improvements in lower-limb strength are essential for activities of daily living such as rising from a chair, climbing stairs, and walking longer distances [24,[48], [49], [50], [51]]. However, these findings did not translate into improvements in muscle mass or physical performance and should therefore be interpreted with caution. Our findings suggest the importance of assessing habitual protein intake when prescribing resistance training in frail older adults. Future research should include larger samples of older adults with low habitual protein intake (0.8 g/kg) to investigate the effects and long-term effects of protein during resistance training on other sarcopenia-related outcomes, with a particular focus on personalized, tailored interventions.

## 5 Conclusion

In conclusion, a protein intervention during resistance training did not result in additional improvements in muscle strength, muscle mass or physical performance in frail community-dwelling older adults. The protein intervention was associated with greater strength gains in exploratory analyses among participants with lower baseline protein intake (<1.2 g/kg/day), with the most pronounced associations observed in those consuming <0.8 g/kg/day. However, these findings were not consistent for muscle mass or physical performance. Training intensity of resistance training performed to muscle failure did not influence the effects of additional protein. These findings should be confirmed in larger trials to validate the observed benefits in frail individuals with low habitual protein intake.

## CRediT authorship contribution statement

**Biersteker**: Methodology, Validation, Formal analysis, Investigation, Resources, Data curation, Writing – original draft, Writing – review & editing, Visualization, Project administration. **Benali**: Conceptualization, Methodology, Investigation, Resources, Data curation, Writing – review & editing, Project administration. **Van den Helder**: Conceptualization, Methodology, Investigation, Resources, Data curation, Writing – review & editing, Project administration. **Twisk**: Methodology, Validation, Formal analysis, Writing – review & editing. **Tieland**: Conceptualization, Methodology, Writing – review & editing. **Weijs**: Conceptualization, Methodology, Writing – review & editing, Supervision. **Schoufour**: Conceptualization, Methodology, Validation, Writing – original draft, Writing – review & editing, Supervision.

## Declaration of Generative AI and AI-assisted technologies in the writing process

During the preparation of this work the authors used ChatGPT (AI language model) in order to help with grammar, spelling, and sentence structure. No analytical or interpretative content was produced by the model. After using this model, the authors reviewed and edited the content as needed and take full responsibility for the content of the publication.

## Funding statement

This study is funded by the Taskforce for Applied Research SIA, part of the Dutch Research Council (NWO), grant number RAAK.PRO03.062 and by the Doctoral Grant for Teachers (NWO), grant number 023.020.029. Additional support has been provided by Carezzo Nutrition BV and Fonterra BV through both in-kind and cash contributions. These funders have no influence on the work reported in this paper.

## Data availability

The statistical analysis plan and research protocol will be made publicly and freely available without restriction in a data package on the Figshare repository (https://doi.org/10.21943/auas.29666096). Data described in the manuscript will be made available upon a valid justification.

## Declaration of competing interest

The authors declare that they have no known competing financial interests or personal relationships that could have appeared to influence the work reported in this paper.
